# Duration of Pregnancy

**Published:** 1846-06

**Authors:** 


### DURATION OF PREGNANCY.

We extract the following account of legal proceedings in a case of bastardy, from the American Republican, published at Lancaster, Pa., of the 2d of May, 1846.

"*An Important Law Case.*—The following report of an important case, tried in the Quarter Sessions of this county, last week, was originally prepared by a gentleman of this city for his own use, from memory. Of course the exact words of the witnesses are not given, but it is believed that it will be found correct in every material point. There may be, we are aware, some persons who will object to the publication of such reports, but the importance of the case we think will prove an ample justification, it being, so far as we can learn, in one of its features, unprecedented in the United States.

| | |
|---|---|
| *Commonwealth* vs. *Elijah F. Hoover.* | In the Quarter Sessions of Lancaster county, of April term, 1846. |

This was an indictment for fornication and bastardy upon the body of Catharine E. Reiff. Frazer and Mathiot for the Commonwealth. Stevens for defendant.

The prosecutrix swore that the sexual intercourse took place between 8 and 9 o'clock on the morning of March 23d, 1845, at the house of her father, which is situated but a short distance from the dwelling of the defendant, that the defendant was with her about half an hour or thereabouts, could not tell exactly how long he staid, but knows he went away in time to go to church, three miles off, and that he visited her again the same evening between 11 and 12 o'clock. Knows that it was past 11, because she wondered what kept him so late; was waiting for him. Church goes in at 10 o'clock.

Says she knows a man named Benj. Leaman; never threw her arms around his neck and kissed him; never sat upon his knee; never had any obscene conversation with him. Did lend him a book which had been lying in the bar at her father's, but was unacquainted with its contents, had never read it and did not know what was in it, could not tell whether it had obscene plates or not.

Never went up to her own room at night, and then after the rest of the family were asleep come down again and sit till two o'clock with a strange drover. Never was guilty of such an act in her life. Two or three young men did visit her after the 23d of March, '45, but she did not receive their addresses; did tell one of them to speak to her father about it to get rid of him; did know a man named Z., but did not know where he was now; it was said he was in New York. Never had sexual intercourse with any but defendant. Saw defendant once after March 23d, but had no opportunity to speak to him. My child is a male, and was born on January 30th, 1846. Did not swear before the squire to any other time than the 23d of March. He came to see me for two years.

Eliza Johnson. Says she was present at the birth of the child.

Came about six o'clock in the morning, and remained till nine at night. Prosecutrix said several times that she never thought defendant would bring her to all this misery. It was on the 30th of January last.

A brother of the prosecutrix was offered to prove, that the squire, before whom she had made oath as to the paternity, had told him that the copy of the complaint was wrong, so far as it related to the introduction of the word '*since*' after the words "the 23d of March, 1845, and at other times." The Court overruled the offer, and the testimony was rejected. The squire had been subpœnaed, but being sick could not attend. The docket was then produced and was found to differ in some of the words of the complaint from the copy returned to the attorney general. The word "*since*," upon the inspection of the docket, appeared to have been interlined at a time subsequent to the original entry of the complaint which the prosecutrix had signed.

The defence embraced three grounds; first, an alibi as to both visits; second, that the prosecutrix was of loose character; third, the impossibility of the period of gestation in a healthy case extending to 313 days. To make out the first, the defendant called his father, who testified, that defendant was about all morning and was at home before 9 o'clock; knows the date because he was about to move, and had business to transact of importance near Columbia on the following day; left home on the 23d, a little before or about 9 o'clock. Did not keep his eyes on defendant all morning, but did not miss him from the house at any time before his departure. The distance from my house to the place where prosecutrix lived is about 4 or 500 yards.

A brother of defendant testified that he was at home all morning; helped defendant to feed the cattle; thinks that defendant was not out of his sight more than fifteen minutes at a time that morning. Knows it was the 23d; it was Easter Sunday. The distance between the two houses is about 100 or 120 yards.

An uncle of defendant testified that he did not miss him from home that morning. Did not keep his eye on him the whole time. He might have been away without his knowing of it, but thinks not.

——— testified that he was at the house of —— Henderson on Sunday evening, March 23, 1845. Knows the time, because it was the Sunday after the spring election. Left there about 9 o'clock. Defendant and Wm. Hamilton and others were present. Left them at Henderson's. The distance from Henderson's to the house of prosecutrix' father is about three miles, it may be more, can't tell exactly.

Wm. Henderson testified that on the night of March 23, '45, defendant was at his father's house. Witness left the room a few minutes after eleven o'clock, defendant was there then. Did not go to sleep for half an hour, and defendant could not have gone away while he remained awake without his knowing it; the noise of their horses would have been heard by him. Knows that it was half an hour, because he always lies awake till he goes to sleep. Does not say his prayers; has no other way of telling how long he remained awake than by guessing at it. Knows it was the 23d of March because Hamilton wrote, that evening, the name of defendant with the date in one of my music books. Cannot tell how long I lay awake any other evening one year ago. The distance from our house to Reiff's is about five miles. The book produced, and name and date found as described by witness.

Hamilton, the other person present at Henderson's on the 23d, was not produced; both parties had subpœnaed him, but he was said to be at Havre de Grace, out of the State.

Several other witnesses were examined in relation to this branch of the case, but as their testimony is not material, it is omitted to save room.

B. Leaman sworn: says that he is a married man; that prosecutrix used to come to his house, would sit down on his knee, throw her arms around his neck and kiss him; that on one occasion while out with her hunting blackberries, she asked him several obscene questions, and that she had recommended him to read a book by the Earl of Rochester, full of obscene plates, which book she procured and lent to the witness. Was a tenant of defendant's father up to the first of April, 1845; am now his tenant, having removed on the first of April last to where he now lives.

Susan McCarraher swears, that one Saturday afternoon, about two years ago, she and prosecutrix were walking on the road not far from the house; that two drovers came down the hill riding in a fancy wagon; that one of them jumped out, and that Catharine took his place in the vehicle and rode with one of the drovers to the house, while she walked to the same place with the other. That these drovers remained there till Monday morning, and that on Sunday evening prosecutrix went with her up to her room, but didn't go to bed. She waited till the family were asleep, then went down stairs and sat up with one of the drovers till two o'clock. I did not see her sitting with the drover; she told me so when she came up. She told me not to tell, because her brothers and her father would be angry if they should find it out. Knows the drover was a married man, because the next morning, while making the beds, they looked into his valise and saw and read a letter to him from his wife. I now live with the sister of defendant. It was not me who sat up with the drover. I went to bed.

At a subsequent period of the case, the following testimony was given, which we introduce here because its bearing will be more clearly understood by the reader, than if we should follow the order in which it was given in Court.

The father of prosecutrix testified that on one occasion, but whether it was the time the two drovers were at his house he could not be positive; he overheard Susan complaining to his daughter, because she had left her alone with the stranger. He never saw any thing in the conduct of prosecutrix to make him think she would sit up with a strange drover till 2 o'clock in the morning. Believes her character for truth is unblemished. Defendant was at my house every day for two years.

Four or five witnesses were called who testified that the character of B. Leaman for veracity was bad. They would not believe him on oath. The character of Susan was not impeached by testimony, though her position, as the hired girl of defendant's sister, was commented on by counsel with much severity.

Several witnesses were called, who testified to the good character of prosecutrix; among them three or four respectable young men, who at various times had visited her, and one of whom had made proposals of marriage. They all said her conduct while in their company was irreproachable; that she was a diffident, backward girl, and that with the exception of the matter now trying, her character in all respects was good. Several other witnesses testified to her previous good character, and one or two to the fact, that since the 23d of March, 1845, she had declined to receive the addresses of several young men, having dismissed them on various pretexts. It was also proved that defendant had visited her, as a suitor, for about two years previous to March, 1845. This was all the material testimony given, except the medical evidence."

Delivery having taken place *three hundred and thirteen days* after the time when connection occurred, according to the allegations of the prosecutrix, medical testimony was at this stage of the proceedings adduced, to show the time to which gestation may be protracted, in a normal case. Twelve physicians were examined, all, we believe, residents and practitioners of the city and county of Lancaster—men of experience and reputation in their profession. As usually happens in such cases, the testimony of these gentlemen—or rather, we should say, *opinions*—were exceedingly discordant. Some thought the period was never protracted beyond nine calendar months, or two hundred and seventy days; others, that it might be protracted a few hours or even a few days beyond that; and others, that it was sometimes extended even to ten months. One or more of the latter regarded it as *possible* that gestation might, in a healthy condition of things, be protracted to three hundred and thirteen days, but that it was not very *probable.*

"Both sides having closed the testimony, Mr. Mathiot read the following medical authorities, Hamilton on Midwifery 53 & seq. Dewees 130 & seq. Cyclopedia of Practical Medicine 276 & 281. A case from Moreau's late work on obstetrics. Mr. Frazer read the legal authorities on which he intended to rely.

The medical authority chiefly relied on by the defence was Dr. Beck's Medical Jurisprudence. The case was very ably argued on both sides by Messrs. Stevens and Frazer.

Mr. Mathiot displayed much ingenuity and extensive reading in the examination of the medical witnesses.

The following is the charge of the court as furnished by the Judge to the editor of the Examiner, from which paper of Wednesday last we copy it. Our reporter had prepared a very full synopsis of it, which agrees almost verbatim with the original as published below. This circumstance affords a strong proof that the rest of our report is equally correct.

CHARGE OF THE COURT.

Com. *vs.* Elisha F. Hoover. The defendant is indicted for fornication and bastardy. The prosecutrix, Catherine E. Rieffe, is a competent witness, but her credibility is for the jury. According to her account the child was begotten on the 23d of March, 1845. It was born on the 30th January, 1846—a male, fine, large and healthy. The period of gestation was 313 days. It is conceded that the defendant had no intercourse with the mother after the 23d March, 1845, and the time of delivery is fixed with equal certainty. A question of science has arisen respecting the possibility of protracted gestation.

The usual period is nine calendar months, or from 373 to 375 days. What has been denominated the *extreme* of the *usual period* is 380 days or ten lunar months. But whether, and if any, what longer time may be allowed as possible, are the questions which this case presents for decision. Medical writers of celebrity and authority are arrayed on both sides of these questions. And the medical witnesses upon the stand are in like manner divided in opinion. In construing this evidence, so far as respects the FACTS narrated by each, it is proper to consider that writers and witnesses are respectively relating only the results of their own knowledge; and when one states that no case of protracted gestation has fallen under his observation, it is but negative testimony, and cannot justly be relied upon to invalidate the affirmative evidence of others equally entitled to credit, who enumerate cases of the kind, which they positively affirm to have come within the range of their practice and knowledge. In the most familiar transactions of life, witnesses will differ in their narration of the circumstances. In an account of a simple assault and battery, the bystanders frequently vary in their statement of the facts. Some narrate incidents which others omit. Conceding all the witnesses to be equally worthy of credit, the rule is to reconcile their evidence so that all will stand consistently together, if this be reasonably practicable. Some witnesses observe circumstances which others have not seen. Negative evidence is therefore deemed insufficient to outweigh affirmative statements from witnesses equally entitled to credit. One gentleman, in a long course of practice, may have failed to observe any case of the kind. Another, in a very brief period, may have noticed several. And it is reasonable to believe that where such a diversity of OPINION exists, each will be in some measure influenced by his own professional experience, and that this will also, to some extent, affect his belief in the cases reported by others. There are doubtless many of these cases where the struggle for character and property, and the circumstances of the parties whose interests have been involved, have furnished temptations to falsify, and may have influenced the decisions of the tribunals. But after making all proper allowances for cases of this description, the whole evidence, on the question, when fairly considered, appears to show that cases of protracted gestation are not impossible, although their existence is very unusual.

The heads of wheat in the same field do not all ripen together. The ears of corn on the same stalk do not all come to maturity at the same time. Even the grains of corn on the same ear ripen at different periods. The fruit on the same tree shows the like deviation. A portion will ripen and fall, while other portions remain comparatively green upon the parent stalk. The eggs of the fowl, under process of incubation at the same time, are subject to the same variation. In quadrupeds, if the testimony of M. Teissier be believed, we have proof of the like irregularity. Whatever may be the causes, operating in each case, to divert nature from her accustomed course, to accelerate or delay her usual progress, the human species, like the rest of creation, seem occasionally under their influences. The developments of puberty, although generally shown at a certain age, are far from regular. Some individuals approach it earlier—others later in life. Intellectual maturity is subject to the same irregularities. Some are precocious, others astonishingly tardy in arriving at the usual degree of discretion. The intervals between the catamenial visits, although in general regular and fixed, exhibit remarkable deviations. Their final departure, although generally to be expected at a certain age, are as irregular as their first approaches, and as subject to variation as were their periodical returns. A certain period of life has been usually assigned for the termination of a mother's perils, but the instances of extensive deviations from this general rule are numerous and well established. The gestation of one child at a time, is according to the usual course of nature, but the births of twins, triplets, &c., furnish indubitable proofs of astonishing departures from the usual course. The sensations of the mother produced by the elevation of the fœtus from the cavity of the pelvis (called quickening) although usually occurring at a certain period, are known to be subject to the like departure from the usual time. It has been said that human life does not generally extend beyond 70 years. But if this be the general rule the departures are numerous. The most distinguished jurist perhaps now living in the whole world (Chancellor Kent) will be 83 years old on the 31st of July next; and yet, within a few days, I have been honored by the receipt of a letter from him, under date of the 18th inst., in which he states that he is still in "good and active health—that his relish and ardor for studies and legal learning continue unabated—that he has the blessing of good eyes, and that he is still an observer of what passes with lively sensibility." This instance may serve to illustrate not only the occasional deviation from general rules respecting the duration of human life, but the like variation in respect to intellectual vigor, by which one individual attains a preeminence over the generality of mankind. All nature abounds with occasional departures from her general customs. Even the compass, which guides the mariner on the trackless ocean—which enables science to fix with reasonable certainty the boundaries of kingdoms and farms, and the truthfulness of which to its accustomed law has been perpetuated by a proverb--is subject to mysterious but acknowledged variations.

From analogy, and from the statements of distinguished authors and eminent witnesses, after making every allowance for mistakes, and the operation of unfavorable influences, we are led to the belief that, although Nature delights in adhering to her general usages, she is occasionally retarded in her progress, and otherwise coerced, by causes not always apparent, into extensive deviations from her accustomed path. And we are induced to believe that protracted gestation, for the period of 313 days, although *unusual* and *improbable*, is not *impossible*. The evidence to establish the existence of such a considerable departure from the unusual period, should be clear and free from doubt. The witness should possess a character beyond reproach, and her testimony should be consistent and uncontradicted in all material facts. If the jury are satisfied that the evidence for the Commonwealth is of this character, the unusually long period of gestation does not require them to disregard it. The law fixes no period as the *ultimum tempus pariendi*. The usual period has been stated, but longer time may be allowed, according to the opinions of physicians and the circumstances of the case. The question is, therefore, open for the decision of the jury. If they believe the witness they may find the defendant guilty.

Here the Court drew the attention of the jury to the prominent facts, tending on the one side to impeach, and on the other to support, the credit of the prosecutrix; and then left the case to the jury, with the direction that if they entertained reasonable doubts of the defendant's guilt, he was entitled to an acquittal.

*April* 24th, 1846. ELLIS LEWIS.

The jury found the defendant *guilty*, and the usual sentence was passed."

The weight of authority is certainly with those who admit considerable variations in the duration of pregnancy. Positive facts, and abundant analogy, conclusively establish this point. According to our own experience, the same woman may differ in different pregnancies; while some always anticipate the period of nine months, others constantly exceed it: but that in a natural condition of things it should be extended to *three hundred and thirteen days*, is, we confess, stretching the matter very far. We think it highly probable that the long courtship of the parties, in this case, had not a little influence on the minds of the jury, in arriving at the conclusion expressed by their verdict.—EDITOR.

